# Patient as a Partner in Healthcare-Associated Infection Prevention

**DOI:** 10.3390/ijerph15040624

**Published:** 2018-03-29

**Authors:** Marta Wałaszek, Małgorzata Kołpa, Zdzisław Wolak, Anna Różańska, Jadwiga Wójkowska-Mach

**Affiliations:** 1 State Higher Vocational School, 33-100 Tarnów, Poland; mz.walaszek@gmail.com (M.W.); malgorzatakolpa@interia.pl (M.K.); zdzich_w@interia.pl (Z.W.); 2 St. Łukasz Voivodeship Hospital, 33-100 Tarnów, Poland; 3 Jagiellonian University Medical College, 31-121 Kraków, Poland; rozanska@ifb.pl

**Keywords:** hand hygiene, patient, healthcare workers, healthcare-associated infections

## Abstract

Objectives: The objective of the study was getting to know the knowledge and attitudes towards hand hygiene (HH) among Polish patients and healthcare workers (HCWs). Methods: 459 respondents replied to the survey: 173 (37.6%) patients and 286 (62.3%) HCWs; 57 HCWs were additionally interviewed. Results: Few HCWs knew and used the “5 moments for HH” in the required situations. Both patients and HCWs rated HH of other HCWs poorly: only 75% of patients and 54% of HCWs noticed the application of HH before blood sample collection, but 1/2 of interviewed HCWs did not encounter a request for HH from a patient. According to interviews, 23 (40%) HCWs did not admonish others when they did not use HH. Seventy-five percent of patients and HCWs claimed that, in the past, in schools the toilets were poorly stocked, but the situation improved with the passage of time. Conclusions: There are barriers with resspect to treating patients as partners in HH in Polish hospitals and HCWs’ lack of compliance with the “5 moments for HH” significantly reduces patients’ safety. Practice implications: Education regarding HH should be conducted for the whole society from an early age: lack of proper supplies in school bathrooms impedes the development of positive HH habits.

## 1. Introduction

More and more often, in different countries of the world, patients unite in numerous societies and organizations undertake actions aiming at improving the healthcare safety through their cooperation with healthcare workers (HCWs) [1,2]. These actions also help raise patients’ awareness in the field of prevention of nosocomial infections (HAI) which is needed as patients lacking knowledge concerning hand hygiene (HH) may unwittingly transfer microbes between patients [3]. On the other hand, a patient who is aware of the prophylaxis may become a partner for HCWs by reminding them about the necessity of conducting hand hygiene in certain situations. Initiatives undertaken to improve patient safety include strengthening the patient’s position by including him/her in the self-care process. Generally, patients engage in these activities willingly, but have difficulty drawing HCWs’ attention to hand hygiene as they are apprehensive about their weak position and strong authority on the part of HCWs. Social and cultural factors, as well as deeply-rooted national culture, may reinforce patients’ passivity [4,5,6]. Differences in the perception of hand hygiene and reactions of patients to mistakes in this respect can be attempted to be explained on the basis of cultural dimensions described by Hofstede, i.e., power distance, uncertainty avoidance, individualism/collectivism, and masculinity/femininity [7,8,9,10].

Therefore, one of the objectives of HAI prophylaxis is partnership between HCWs and patients and developing positive habits in patients with respect to hand hygiene, which can be a significant link in achieving this goal. It is an important issue in Poland as previous research indicates substantial deficiencies in the knowledge of hand hygiene among HCWs [11]. An improvement may be reached by directing educational and health-related activities not only to HCWs but, at the same time, to the patients, their families, and local community. Planning educational programs aiming at improving hand hygiene in the society would be possible with the participation of people professionally involved in health activities and after getting to know what the knowledge and attitudes dominant in the local population are.

These reasons justified undertaking our research, the purpose of which is finding out the state of knowledge and attitudes towards hand hygiene among the hospitalized adult patients and HCWs.

## 2. Materials and Methods

The observational and descriptive study was carried out between 1 July–30 September 2015 using the diagnostic survey method in the St. Łukasz multi-profile provincial hospital in Tarnów with 713 hospital beds and 22 hospital departments (nine surgical, 12 non-surgical wards, and one intensive care unit). A specially-devised survey questionnaire (Supplementary Materials) was employed and structured interviews were conducted.

The survey study comprised 459 respondents, including 173 (38%) adult hospital patients and 286 (62%) healthcare workers (HCWs) (Table 1). On 1 July 2015, a random sampling of patients and employees, from the hospital electronic database, was carried out. From the group of 629 patients and 846 HCWs, two samples of 300 subjects each were drawn. The drawing was carried out using a standardized table of random numbers. Each patient and HCW had an equal opportunity to be drawn. The return response rate in case of patients was 58% (173 questionnaires) and, in the case of HCWs, 95% (286 questionnaires). The questionnaire was not filled out by 127 (42%) patients. The lack of answers usually resulted from poor health, which made it impossible to fill in the questionnaire, or from refusal to take part in the study.

1. In the group of 173 studied patients, there were 121 (70%) women and 52 (30%) men; 90 people (52%) lived in the countryside and 83 (48%) in urban areas. The examined patients represented different levels of education: 50 (29%) had vocational education, 65 (38%) completed their education at the secondary level, and 29 (17%) had a university degree (29 people, 17%—no data). The distribution of the type of patients’ education (profile) was as follows: 21 (12%) patients had higher medical and related education (like biology), 46 (27%) had humanistic education, and 43 (25%) had technical education (63 people, 36%—no data). The largest group (72 people, 42%) were patients aged <41 years, next (60, 35%) were those 41–60 years, and the smallest group (41, 24%): 61 years or more.
2. In the group of 286 HCWs, there were 185 (65%) nurses, 47 (16%) intern physicians, 39 (14%) medical students, and 15 (5%) nursing students. Among the studied staff, there were 251 (88%) women and 35 (12%) men. The work experience was very diverse: 81 persons (28%) worked <6 years, 72 (15.6%) were 6–20 years, and 134 (29%) were more than 20 years (Table 2). Healthcare workers (HCWs) were selected at random. For medical students and nursing students, target screening was used: they were examined on the first day of their professional practice and came from different medical universities located in Poland. The research was expanded to include interviews (qualitative research) with HCWs, who consented to this form of examination.
3. The interviews were conducted with 57 HCWs: 19 (33%) nurses, 12 (21%) intern physicians, 11 (19%) medical students, and 15 (26%) nursing students. With the consent of the subjects studied, the interviews were recorded, and then transcripts were prepared. During the interviews, the principles of kindness, intimacy, universalism, and impartiality were observed.

HCWs’ knowledge in the field of “5 Moments for HH” was tested by the questionnaire. The concept of “5 Moments for HH” merges the HH indications recommended by the WHO Guidelines on HH in Health Care [12] into five moments when hand hygiene is required: (1) before touching a patient; (2) before clean/aseptic procedure; (3) after body fluids exposure risk; (4) after touching a patient; and (5) after touching patient surroundings. The question asking ‘when should the 5 Moments for HH be performed’ was open, and here the respondents—only HCWs—could give no more than five answers on their own, which were then statistically elaborated, as an analysis of multiple responses. The remaining questionnaire questions employed the Likert scale or the option of choosing the response, ‘*yes*’ or ‘*no*’ (Supplementary Material).

Among the patients, the variables tested were: age, education level and type, place of residence, and among medical personnel: seniority and place of work. In the statistical analysis of the material collected, statistical software IBM SPSS (SPSS—Statistical Package for the Social Sciences) STATISTICS 24 (IBM, Armonk, NY, USA) and Microsoft Excel (Microsoft Office 2016, Redmond, WA, USA) were employed. The description of data concerning the whole population studied was prepared with the use of mean values and 95% confidence interval for the mean. In order to compare the groups studied in terms of the frequency of occurrence of variants of qualitative characteristics, the Pearson’s chi-squared test of independence (*p*) was used and ANOVA was employed for quantitative features. Pearson’s correlation coefficients (R) were also calculated for the description of the relationship of the variables studied.

The use of data was approved by the Bioethical Committee of the Jagiellonian University (no. KBET/122.6120.124.2016).

## 3. Results

### 3.1. Survey Questionnaire Results

In one of the survey questions, healthcare workers (HCWs) were asked to list all “5 moments for HH”. In response to this question, as many as 171 (60%) HCWs could not name the point ‘*before patient contact*’, 158 (55%) did not list ‘*before aseptic task*’, 244 (85%) failed to mention ‘*after body fluid exposure risk*’, 177 (62%) forgot about ‘*after patient contact*’, and 281 (98%) did not list ‘*after contact with patient surroundings*’. Among the 286 HCWs studied, 192 (66.8%) knew at least one of the “5 moments for HH”, but as many as 94 (33%) did not know any of the “5 moments for HH”. Medical workers with seniority under 6 and over 20 years were more likely to list the “5 moments for HH” properly (Table 2).

The examined patients were asked the following question: *Being at the hospital, were you aware that the medical staff were performing HH in the course of the activities associated with medical procedures*? A vast majority, i.e., 127 (73.4%), of the patients studied declared that they saw the personnel disinfect their hands prior to procedures performed on them, 14 (8.1%) did not see them, and as many as 32 (18.5%) of the patients abstained from answering the question. The patients stated that in approx. 40% of cases, the staff disinfected their hands ‘always’ and ‘often’, but for around 20% of the situations, it happened rarer before direct physical contact or physical examination. Other observations concerned drawing blood, the second point of the “5 moments for HH”, were that 75% of patients ‘always’ saw hand disinfection of HCWs and 22%—did it ‘often’ (Table 3). A more extensive analysis of this issue considering patients’ professional education demonstrated differences in terms of observing hand disinfection prior to drawing blood. Patients with medical and related education were more critical of the activities of the medical staff and noticed that their hands were not always disinfected, although the situation made this action necessary. Only 58% of patients with medical and related education confirmed that the medical workers ‘always’ disinfected their hands before piercing a patient’s vein, which was confirmed by 90% of patients with humanistic education and 71% of patients with technical education (*p* < 0.05).

The responses of medical workers as regards the observed hand disinfection in this situation (with respect to their own experiences from outside of work), who answered the question: *Did you observe that HCWs performed HH before drawing your blood for testing?* in 53% confirmed that they ‘a*lways*’ witnessed HH, then, 37% answered ‘*often*’ and 10% ‘*rarely*’—the results were similar to the answers of patients with medical (and related) education. The same situation was evaluated differently by HCWs.

Both the patients and HCWs were asked: *When you were in primary and secondary school, were there towels and soap in the school bathrooms?* The vast majority of the respondents (329; 71.2%) replied that their school did not provide such supplies. Only 105 (22.7%) people declared that the school which they attended had soap and towels, 28 (6.1%) subjects did not provide an answer. The answers given to this question did not demonstrate statistical differences between patients and HCWs (R = 0.056; *p =* 0.242); patient education (R = −0.064; *p =* 0.638); patient place of residence (R = −0.029; *p* = 0.718); gender of HCWs (R = 0.059; *p* = 0.329); seniority of HCWs (R = 0.120; *p* = 0.125); or professional groups of HCWs (R = 0.001; *p* = 0.852). Patients with humanistic education more often than others signaled that the school washing stations are sufficiently equipped (R = 0.166; *p* < 0.001). Patient age analysis showed that there was a significant improvement of school bathroom supplies regarding soap and towels in the opinion of respondents aged 20–40 years (41.9% of positive ratings), 41–60 years (27.1%) and 61 years and older (2.8% of positive ratings) (R = 0.326; *p* < 0.001) (Table 4).

Both HCWs and the patients were asked in the survey about the level of hand hygiene that they felt was necessary after contamination in particular situations associated with health and disease (Likert scale: 1—clean hands, 7—very dirty hands). Statistically significant differences were demonstrated in the perception of the mentioned situations: HCWs felt the need for hand hygiene significantly more. Only shaking hands and washing oneself were evaluated similarly by both the patients and HCWs (Table 5).

### 3.2. Interview Results

In interviews, HCWs were asked to assess the hand hygiene level of other HCWs on the scale of 1 to 5. Hand hygiene of other HCWs received the highest ratings from medical students (mean of 3.9) and the lowest from nurses (2.9), however, the scores from intern physicians (3.1) and nursing students (3.0) were similar to the ones from nurses, and the differences were statistically significant (*p* < 0.01) (Table 6).

When replying to the question concerning the reaction to the situation of a patient admonishing the HCW for lack of HH, most of the interviewed people (53.6%) responded by saying that such a situation had never taken place. The remaining subjects, who had had such an experience, felt great discomfort, shame, embarrassment (20, 35%); some of the studied people (8.9%) approached the problem in a task-oriented way and replied, ‘*I performed HH*’; isolated responses revealed aggressive and defensive reactions, ‘*I convinced the patient that my hands are clean*’, ‘*it was an attack on me*’ (Table 3). In response to the question, ‘*when would you draw somebody’s attention to the fact that they do not comply with HH*’, the respondents usually said that would not admonish someone with a higher rank (10, 18%), they would not be brave enough (9, 16%), 2 (4%) had bad experiences, and 1 (2%) claimed that students cannot reprimand other people.

## 4. Discussion

The investigation of the knowledge and skills of HCWs may help to identify the educational areas that have to be implemented. The study presented by us finds a relatively poor knowledge concerning HH among the personnel. The results of epidemiological studies in Poland confirm that the compliance with HH is not sufficiently kept by HCWs, which is evidenced by the findings on the horizontally-transmitted microorganisms in hospital outbreaks in Poland, especially in adult and neonatal ICUs [13,14]. In this research work, there were significantly worse results concerning the knowledge of the “5 moments for HH” presented by workers with medical seniority, which can be closely associated with the time when the WHO idea first appeared and, hence, more senior HCWs might not have had it as part of their formal education. Our data also show the importance of postgraduate education for older employees who know HH rules. Unfortunately, according to the prior experiences of the authors, the subject of HH is skipped in the course of vocational training: professional practice of 22.9% of the studied respondents—medical staff in Polish hospitals—was not preceded by any training in the field of hospital hygiene and in 28% of cases training did not cover HH [15].

Only a half of the HCWs studied by us knew that another HCW had disinfected their hands prior to drawing blood for testing. Lack of such observations in the remaining cases may suggest poor compliance with the rule of ‘*hand disinfection before aseptic procedure*’ and is confirmed in the answer to the question about “5 moments for HH”, in which only 60% of the HCWs studied could name this moment of hand hygiene. It all points to substantial knowledge deficits among HCWs in relation to the “5 moments for HH”. Additinoally, in the parallel question, HCWs declared in only 53% that they ‘*always*’ saw hand disinfection before their blood was taken when they were patients themselves. The answers of patients with medical/related education were very similar, as only 58% of these patients confirmed that they witnessed hand disinfection before taking blood samples. Patients with humanistic and technical education confirmed the disinfection of hands before blood collection more often. Lower scores of HCWs and patients with medical education stem from a greater medical awareness, therefore, HCWs, as well as people with a background in medicine (or in general, nature) pay attention to the aspects of prophylaxis and have a more rigorous opinion with respect to the hand hygiene practices employed in the medical area. It also means that more than half of patients may have been exposed to the transmission of infection during aseptic tasks. Różańska et al. [16] demonstrated that hospital surfaces can be heavily colonized with microorganisms, and contact of HCWs’ hands with these surfaces results in their contamination and, consequently, the possibility of transferring microorganisms to the patient. However, HH only may be insufficient; for example, it has been shown that drying after washing hands is a necessary phase in pre-operative preparation. Therefore, hand hygiene must be part of a more complex strategy of nosocomial infection control [17].

A considerable number of the patients studied knew that HCWs disinfected their hands for them prior to performing a given procedure, which may suggest that a vast majority of patients show interest in hand hygiene during their hospital stay. Admittedly, 1/5 of the patients abstained from answering the question, which may be connected with an actual lack of performing HH by HCWs. In the study by Sunkesula et al. [18], it was proved that the majority of patients were aware of the significance of hand hygiene in the prevention of infections. Ardizzone et al. [19] described how to effectively include a patient into the HH activity in a surgical unit. Unfortunately, in the study by Lent et al. [20], the hospitalized patients, despite encouragement, were not always inclined to ask HCWs to perform HH and they did not pay attention to omitting hand disinfection during rounds [21]. Butenko et al. [4], while doing a systemic review, discovered that the organizational culture allows to include the patient into the process of improving safety during treatment, however, the national culture, beliefs, and behaviors of HCWs do not fully support this partnership. Patients also have varying levels of knowledge and sensitivity in HH and may be afraid of unfavorable relations after admonishing the HCWs [4]. Scheithauer et al. [6] observe that, at the implementation stage of HH improvement projects, a multi-faceted knowledge of problems taking into account social and cultural conditions is essential. The presented data, in which only 1/10 of HCWs disinfected their hands after being admonished by a patient, and over a half did not give an answer to the question concerning their reaction to the patient’s remark about HH, seem to indicate that, in Poland, the patient is expected to be passive in this respect. Such a passive attitude to HH shortcomings stems from the national culture, in which power distance and uncertainty avoidance blocks partnership [7,8,9,10]. Hence, particularly in Poland, health-promoting campaigns should be conducted locally and in a manner adapted to the specific living conditions, as this is when they are most efficient [21].

In the differences which we showed here as regards the perception of feeling ‘*dirty*’, patients more often than HCWs perceived the analyzed situations as ‘*clean*’. Consequently, it may be rarer for patients to feel the need to conduct hand hygiene of their own hands during the hospital stay. Both patients and HCWs had a high feeling of dirt in contact with a sick person and visits to the hospital. The level of ‘*cleanliness and dirt*’ felt can translate to decisions concerning whether to perform HH or not. The described diversity in the perception of cleanliness and dirt in the medical field points to a need for education and supporting the patient in the pursuit of raising awareness and building positive health habits.

In this study, over 70% of the subjects (patients and HCWs) stated that, in the course of their education, the schools which they had attended did not provide appropriate supplies to conduct hand hygiene: the older the respondent, the lower the availability of soap and towels in the bathroom. Fortunately, this research simultaneously indicates that the situation is getting better and the youngest patients assessed the hygienic conditions in their schools slightly better. However, in contrast to the investigated sample, the most often hospitalized people in Poland are aged 65+, and they constitute 34.1% of all hospitalized people in total with the indicator of 41.7/100 residents in 2015, and with total hospitalizations of 18.9/100 residents, which means that, in the Polish hospital, an HCW generally deals with people who have a low hygienic capital [22].

That is why it is necessary to tighten sanitary requirements in healthcare and public use facilities aiming at early training in rules of hygiene and promotion of positive models of infection prevention. Of particular importance are the actions referring to the growing microbial resistance to antibiotics, where it was proved that contaminated patient hands may contribute to the spread of these microorganisms [23].

This study has some limitations. First of all, there was no pilot study performed on a smaller sample in order to identify questions that might raise doubts or misunderstanding. However, the questions, especially those aiming to identify patient and HCW characteristics were rather clear. Additionally, we did not examine in depth the reasons of given attitudes towards hand hygiene in the group of patients and HCWs. However, our results indicate the possible influence of general education aiming at broad aspects of hygiene and compliance of HH in healthcare settings.

As is clear from the presented study, hand hygiene in Poland is based on a weak foundation, but it also shows slow positive changes in HH and infection prevention and the trend of improving the standards of HH management in the young generation both among patients, as well as HCWs. This is good news, because the saying ‘*As the twig is bent, so grows the tree*’ is not anything new.

## 5. Conclusions

1. The level of knowledge and compliance with hand hygiene in terms of the “5 moments for HH” is insufficient among both the Polish HCWs, as well as patients. Additionally the Polish HCWs generally deal with people who have a low hygienic capital.
2. There is great difficulty in accepting the patient as a partner who can remind one of hand hygiene.
3. There are significant differences between patients and medical staff with respect to feeling the need for carrying out hand hygiene in various health and disease situations.
4. Education on HH has to be conducted for the whole society from an early age until old age.

## Figures and Tables

**Table 1:** Participants of the study divided into surveyed questionnaires and interview questionnaires.

Studied Persons n (%)	Survey Study	Interviewed
patients	173 (38%)	0 (%)
HCWs:		
nurses	185 (65%)	19 (33%)
intern physicians	47(16%)	12 (21%)
medical students	39 (14%)	11 (19%)
nursing students	15 (5%)	15 (27%)
Total HCWs	286 (62%)	
Total (patients and HCWs)	459 (100%)	57 (100%)

**Table 2:** Answers to the open question “when should the “5 Moments for HH” be performed?”, taking into account the age of the respondents and HCW professional groups.

5 Moments for HH:	Seniority of HCWs: n (%)			Professional Groups n (%)			Correct Answers n (%)
	<6 Years N = 81	6–20 Years N = 72	>20 Years N = 133	Physicians N = 47	Nurses N = 185	Students N = 54	
(1) before touching a patient	42 (21.6)	30 (15.4)	43 (22.1)	20 (10.4)	65 (33.2)	30 (15.5)	115 (59.2)
(2) before clean/aseptic procedure	42 (21.6)	32 (16.4)	54 (27.8)	31 (16.1)	71 (37.3)	26 (13.5)	128 (66.9)
(3) after body fluids exposure risk	18 (9.2)	6 (3.1)	18 (9.3)	8 (4.1)	21 (11.4)	13 (6.7)	42 (21.6)
(4) after touching a patient	38 (19.6)	33 (17.0)	38 (19.6)	17 (8.8)	64 (32.6)	28 (14.5)	109 (56.2)
(5) after touching patient surroundings	1 (0.5)	1 (0.5)	3 (1.5)	0 (0.0)	3 (2.1)	2 (1.0)	5 (2.5)
total	63 (32.4)	50 (25.7)	81 (41.7)	36 (18.7%)	114 (59.1%)	43 (22.3%)	

Healthcare workers (HCWs); hand hygiene (HH), HCW number (N). n—number of correct answers for each point of “5 Moments of HH” in given respondent categories.

**Table 3:** Summary of the answers of patients and HCWs arising from the question: How often did HCWs perform HH before first contact, touching you, medical examination, administering oral medication, or piercing a vein?

Situations Requiring the Performance of Hand Disinfection by HCWs	Always n/%	Often n/%	Rarely n/%	Total n/%	No Answer * n
**Patients n = 173**					
Before touching	51 (40%)	56 (43%)	22 (17%)	129 (100%)	44
Before medical examination	56 (44%)	50 (39%)	22 (17%)	128 (100%)	45
Before administering oral medication	44 (39%)	41 (37%)	27 (24%)	112 (100%)	61
Before drawing blood/piercing the vein	99 (75%)	29 (22%)	4 (3%)	132 (100%)	41
**HCWs n = 286**					
Before drawing blood/piercing the vein	152 (54%)	106 (38%)	21 (8%)	279 (100%)	7

Frequency (n), percentage (%), healthcare workers (HCWs), hand hygiene (HH), * patients who did not answer the question were excluded from the analysis (no answer).

**Table 4:** A summary of patients’ answers divided into education, age, and gender.

When you were attending primary and secondary schools, was there a supply of soap and towels in the bathrooms?				
education	Yes n (%)	No n (%)	Total n (%)	No answer *
medical and related	3 (15.0)	17 (85.0)	20 (100)	1
humanistic	24 (58.5)	17 (41.5)	41 (100)	5
technical	4 (10.8)	33 (89.2)	37 (100)	6
Total	31 (31.6)	67 (68.4)	98 (100)	12
Greatest credibility Chi2 = 25.815 (4); *p* < 0.001; The Spearman’s correlation = 0.166				
age (years)	Yes n (%)	No n (%)	Total n (%)	No answer *
20–40	26 (41.9)	36 (58.1)	62 (100)	10
41–60	16 (27.1)	43 (72.9)	59 (100)	1
>60	1 (2.8)	35 (97.2)	36 (100)	5
Total	43 (27.4)	114 (72.6)	157 (100)	16
Greatest credibility Chi2 = 36.069 (6); *p* < 0.001; The Spearman’s correlation = 0.326				
gender	Yes n (%)	No n (%)	Total n (%)	No answer *
Female	35 (32.7)	72 (67.3)	107 (100)	14
Male	8 (16.0)	42 (84.0)	50 (100)	2
Total	43 (27.4)	114 (72.6)	157 (100)	16
Greatest credibility Chi2 = 10.224 (6); *p* = 0.115; The Spearman’s correlation = 0.094				

* patients who did not answer the question were excluded from the analysis (no answer).

**Table 5:** The level of the need for hand hygiene felt after the occurrence of contaminated hands between patients and HCWs in particular situations associated with health and disease, on a scale of 1 (clean hands) to 7 (very dirty hands).

Situations	Patient	HCWs	*p*-Value
	Mean (95% CI)	Mean (95% CI)	
Contact with secretions	5.6 (5.4–6.1)	6.4 (6.2–6.5)	<0.001
Removal of impurities	5.0 (4.6–5.4)	5.6 (5.7–6.1)	<0.002
Change of diaper pants	5.0 (4.7–5.4)	5.8 (5.4–5.8)	<0.003
Contact with patient	4.5 (4.2–4.9)	4.8 (4.5–4.9)	0.067
Shaking hands	4.2 (3.8–4.4)	4.0 (3.8–4.2)	0.192
Nursing the patient	4.1 (3.7–4.3)	4.8 (4.4–4.9)	<0.001
Visiting the hospital	4.0 (3.7–4.3)	4.4 (4.2–4.7)	<0.01
Hospital stay	3.6 (3.2–3.9)	4.6 (4.4–4.8)	<0.01
Washing feet	3.1 (2.7–3.4)	3.7 (3.4–3.8)	<0.01
Doctor’s appointment	2.9 (2.4–3.0)	3.4 (3.2–3.6)	<0.01
Surgical procedure	2.6 (2.2–2.8)	3.1 (2.9–3.4)	<0.01
Washing your own body	2.5 (2.1–2.9)	2.8 (2.4–2.9)	0.204

95% confidence interval for the mean (95% CI), ANOVA (*p*) significance; healthcare workers (HCWs).

**Table 6:** Subjective rating on a scale of 1 to 5 of the observed level of hand hygiene of other HCWs and HCW attitudes towards problems associated with compliance with the rules of hand hygiene.

Professional Group	Mean (95% CI)
intern physicians (a scale of 1 to 5)	3.1 (2.5–3.7)
nurses (a scale of 1 to 5)	2.9 (2.7–3.1)
medical students (a scale of 1 to 5)	3.9 (3.1–4.7)
nursing students (a scale of 1 to 5)	3.1 (2.6–3.2)
**Total (a scale of 1 to 5)**	**3.2 (2.9–3.3)**
*How did you feel when a patient admonished you for not complying with HH?*	**n (%)**
discomfort, shame, embarrassment	20 (35.1)
I performed disinfection	5 (8.8)
It was an attack on me, I argued that my hands are clean	2 (3.5)
No answer	30 (52.6)
*Did you draw attention of HCWs to the fact of not complying with HH?*	**n (%)**
no	23 (40.4)
yes	8 (14.0)
no answer	26 (45.6)

n (number), 95% confidence level for the mean (95% CI), healthcare workers (HCWs), hand hygiene (HH).

## Supplementary Materials

The following are available online at http://www.mdpi.com/1660-4601/15/4/624/s1.

## Abbreviations

HAI	Healthcare-associated infections
HCW	Healthcare worker
HH	Hand hygiene
