# Supplementary material for: Patient as a Partner in Healthcare-Associated Infection Prevention
Source: Int J Environ Res Public Health. 2018 Mar 29;15(4):624. doi: 10.3390/ijerph15040624 (PMC5923666; doi:10.3390/ijerph15040624)
Supplement: Supplementary file 1 [file ijerph-15-00624-s001.pdf]

Supplementary Material

Annex 1. Questions used in the questionnaire survey and in structured interviews (hand hygiene, HH, healthcare workers, HCWs).

**Questionnaire Survey—Questions About the Knowledge of Hand Hygiene**
1.1. Questions that only HCWs were asked:
When should the '5 Moments for HH' be performed?(open question)
How often other HCWs performed HH before drawing your blood for testing (in outside analytical laboratories)? (Likert scale: always/ often/ rarely)
Would you admonish an HCW that they do not comply with HH? (answer choice: yes/no)
1.2. Question that only patients were asked:
Being at the hospital, did you know that the medical staff conducted HH during medical procedures? (answer choice: yes/no)
How often did HCWs perform HH before touching you? (Likert scale: always/ often/ rarely)
How often did HCWs perform HH before examining you? (Likert scale: always/ often/ rarely)
How often did HCWs perform HH prior to oral drug administration? (Likert scale: always/ often/ rarely)
How often did HCWs perform HH before drawing blood / piercing a vein? (Likert scale: always/ often/ rarely)
1.3. Questions that both HCWs and patients were asked:
When you were attending primary and secondary schools, was there a supply of soap and towels in the bathrooms? (answer choice: yes/no)
Give the level of the felt need for hand hygiene after their contamination in particular situations associated with health and disease (Likert scale: 1 – clean hands, 7 – very dirty hands)
**Structured Interviews—Questions (only HCWs)**
On a scale of 1 to 5, how do you assess the level of HH among HCWs? (Likert scale: 1 – Very Low; 5 – Very High)
What did you feel when a patient admonished you for not complying with HH?
Have you ever drawn the attention of an HCW because of a lack of conducting HH? (answer choice: yes/no) and in what circumstances?
